# A first genetic portrait of synaptonemal complex variation

**DOI:** 10.1371/journal.pgen.1008337

**Published:** 2019-08-26

**Authors:** Richard J. Wang, Beth L. Dumont, Peicheng Jing, Bret A. Payseur

**Affiliations:** 1 Laboratory of Genetics, University of Wisconsin-Madison, Madison, WI, United States of America; 2 Department of Biology, Indiana University, Bloomington, IN, United States of America; 3 The Jackson Laboratory, Bar Harbor, ME, United States of America; The University of Texas MD Anderson Cancer Center, UNITED STATES

## Abstract

The synaptonemal complex (SC) is a proteinaceous scaffold required for synapsis and recombination between homologous chromosomes during meiosis. Although the SC has been linked to differences in genome-wide crossover rates, the genetic basis of standing variation in SC structure remains unknown. To investigate the possibility that recombination evolves through changes to the SC, we characterized the genetic architecture of SC divergence on two evolutionary timescales. Applying a novel digital image analysis technique to spermatocyte spreads, we measured total SC length in 9,532 spermatocytes from recombinant offspring of wild-derived mouse strains with differences in this fundamental meiotic trait. Using this large dataset, we identified the first known genomic regions involved in the evolution of SC length. Distinct loci affect total SC length divergence between and within subspecies, with the X chromosome contributing to both. Joint genetic analysis of MLH1 foci—immunofluorescent markers of crossovers—from the same spermatocytes revealed that two of the identified loci also confer differences in the genome-wide recombination rate. Causal mediation analysis suggested that one pleiotropic locus acts early in meiosis to designate crossovers prior to SC assembly, whereas a second locus primarily shapes crossover number through its effect on SC length. One genomic interval shapes the relationship between SC length and recombination rate, likely modulating the strength of crossover interference. Our findings pinpoint SC formation as a key step in the evolution of recombination and demonstrate the power of genetic mapping on standing variation in the context of the recombination pathway.

## Author summary

During the first stages of meiosis, the chromosome axes are organized along a protein scaffold in preparation for recombination and their subsequent segregation. This scaffold, known as the synaptonemal complex (SC), is critical for the regular progression of recombination. A complex relationship exists between the organization of the SC, the frequency of recombination, and the likelihood of improper chromosome segregation. In this study, we investigate the genetics of synaptonemal complex variation in the house mouse and connect it with variation in the rate of recombination. We found five loci and several compelling candidate genes responsible for the evolution of synaptonemal complex length within and between mouse subspecies. Several of these loci also affect recombination rate, and our joint analyses of the phenotypes suggest an order by which their effects manifest within the recombination pathway. Our results show that evolution of SC length is crucial to recombination rate divergence. Our work here also demonstrates that genetic analysis of additional meiotic phenotypes can help explain the evolution of recombination, a fundamental evolutionary force.

## Introduction

In most species that reproduce sexually, homologous chromosomes must undergo recombination to segregate properly during meiosis [1–4]. Recombination diversifies offspring genomes, shaping evolution and genomic patterns of variation in populations [5–7]. Despite these functional roles for recombination, its frequency varies markedly—on both an evolutionary scale (within and between species), and across genomic scales (ranging from kilobases to chromosomes) [8–12]. This variation has implications for human health: too few or too many crossovers can lead to infertility, miscarriage, or birth defects [3,13,14].

Significant progress on two fronts is laying the foundation for discovering mechanisms responsible for recombination rate differences between individuals. First, work with genetic model organisms is revealing in increasing detail the molecular and cellular processes that lead to crossovers [15–18]. Second, genes and genomic regions that confer standing differences in recombination rate among individuals are being identified through association or linkage mapping [19–32].

Heritable variation in recombination rate is caused by mutations affecting one or more of the steps in the recombination pathway that culminate in crossover formation. Consequently, genetic dissection of inter-individual differences in these formative processes should be especially revealing about how recombination rate evolves. A promising intermediate phenotype to target for such investigations is chromosome synapsis. Synapsis between homologous chromosomes is mediated by the synaptonemal complex (SC), a meiosis-specific supra-molecular protein structure [33,34]. Synapsis and the SC are intimately linked to crossovers. Formation of the SC begins at sites of programmed double-strand breaks (DSBs) early in meiosis [35–37]. In several species, these DSB sites have been shown to mediate the homology search that precedes chromosome pairing; as meiosis progresses, a small subset of DSBs are repaired as crossovers [18,38,39]. During synapsis, the DNA in each chromosome is organized into an array of loops with the SC serving as the central axis that maintains the tight alignment among homologs [40,41]. Mutations that affect SC structure distort recombination patterns, in addition to chromosome pairing and segregation [42–47].

The length of the SC axis is a quantitative characteristic of synapsis that is strongly associated with recombination rate. Variation in SC length reflects differences in the degree of chromatin compaction and interaction between homologs [48–50]. Environmental factors, including temperature, can simultaneously alter SC length and the frequency of recombination [51,52]. In humans, both SC length and crossover number are higher in oocytes than in spermatocytes [53,54]. More broadly, SC length and recombination rate are correlated across multiple mammalian species [55–57], suggesting that variation in SC length and variation in crossover number share a common genetic basis. While the SC has been studied from a mechanistic perspective [33,34,50], the genetic basis of standing variation in this fundamental meiotic phenotype remains unknown.

**Fig 1:**
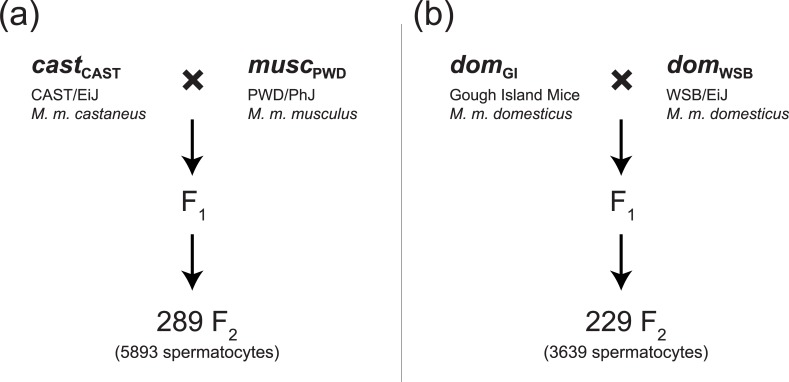
Cross design. Diagram of the intercross design using wild-derived strains (a) between subspecies of *M*. *m*. *musculus* and *M*. *m*. *castaneus*, represented by *cast*_CAST_ and *musc*_PWD_, and (b) within subspecies of *M*. *m*. *domesticus*, represented by *dom*_GI_ and *dom*_WSB_. F_2_s in both crosses are a mix from reciprocal cross directions. The abbreviations for each strain in bold are used throughout.

To fill this notable gap, we combined a novel high-throughput digital image analysis technique and immunofluorescent cytology with the power of complex trait mapping. Genetic dissection of SC length variation in thousands of spermatocytes taken from two mouse intercrosses reveals genomic regions responsible for the evolution of SC structure. These two intercrosses (Fig 1) represent divergence on two different timescales: one between lines diverged by over 300,000 years [58], referred to here as a cross between subspecies, and one between lines that diverged much more recently [59], referred to here as a cross within subspecies. By combining the genetic analysis of SC length and crossover number in individuals from the same dataset, we uncover important evolutionary and genetic connections, including evidence for a common genetic mechanism underlying variation in these two meiotic traits.

## Results

The SC is composed of two lateral elements, a central element, and the transverse filaments that connect them [4,60–62]. At the pachytene stage of meiosis, SC assembly is complete and chromosomes undergo homologous recombination. We used immunofluorescent microscopy to visualize spermatocytes stained with antibodies to SYCP3—a component of the lateral element of the SC—to identify pachytene cells and to measure SC axis length. We calculated a total SC length for individual spermatocytes by summing the axis lengths from each of their 19 autosomes, plus the entirety of the X and Y chromosome axes. The mean of this total length across spermatocytes from each individual was treated as a trait value for genetic mapping in two separate intercrosses between wild-derived strains of house mice (Fig 1): (1) PWD strain (subspecies *M*. *m*. *musculus*; subsequently referred to as *musc*_PWD_) x CAST strain (subspecies *M*. *m*. *castaneus*; *cast*_CAST_), and (2) Gough Island strain (subspecies *M*. *m*. *domesticus*; *dom*_GI_) x WSB strain (subspecies *M*. *m*. *domesticus*; *dom*_WSB_). These strains were chosen to capture genetic variation between and within subspecies, and to examine SC length in crosses for which we had already characterized the genetics of genome-wide recombination rate. To jointly map loci that affect SC length and recombination rate, we combined newly generated SC length data with counts of MLH1 foci (immunofluorescent markers of crossovers) from the same mice reported in [25] and [63].

### Digital image analysis reliably measures SC lengths

**Fig 2:**
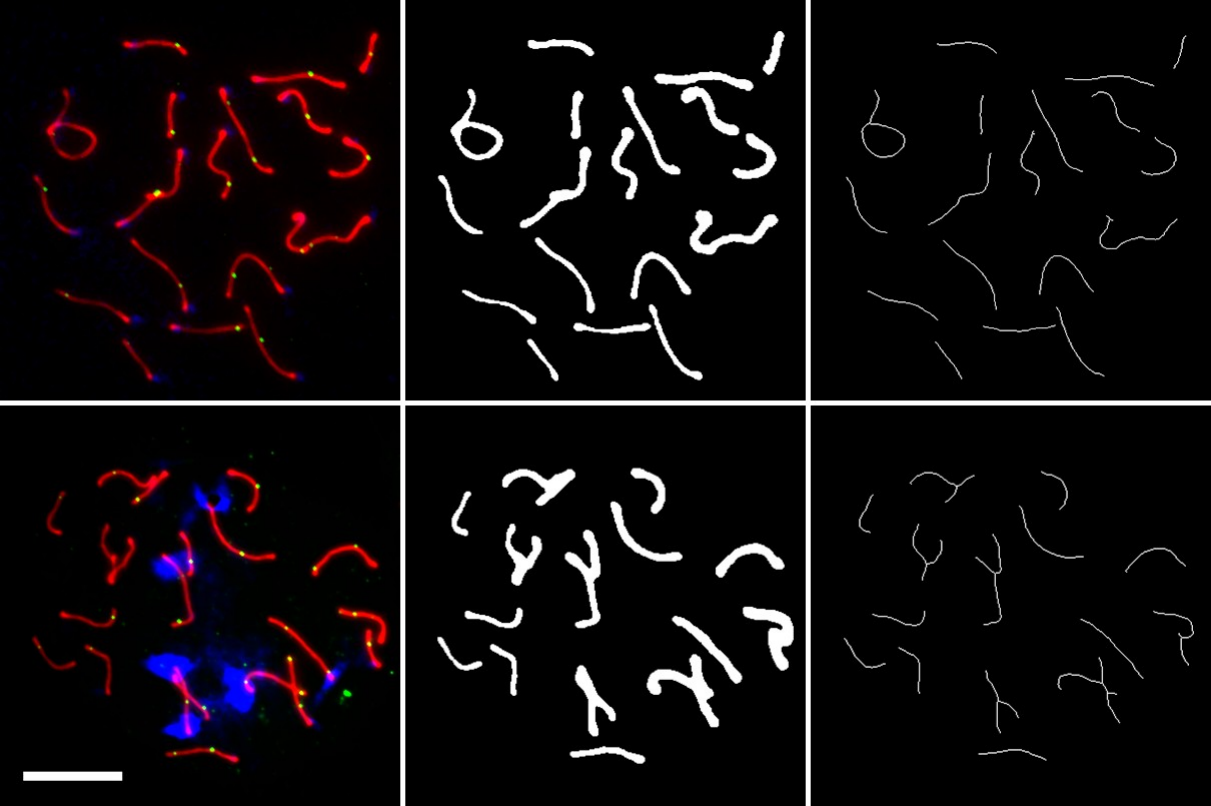
Progression of spermatocyte images through image analysis. On the left, cytological images of a pachytene spermatocyte from a *cast*_CAST_ × *musc*_PWD_ F_2_ (top) and a *dom*_GI_ × *dom*_WSB_ F_2_ (bottom). Chromosome spreads stained for SYCP3 in red, MLH1 in green, and DAPI in blue. Scale bar = 10μm. The respective images after isolating, balancing, and thresholding the red channel (middle); and their single-pixel-wide representation after applying the skeletonize algorithm (right).

To characterize the SC in 9,532 spermatocyte images, we developed a digital image analysis algorithm that automatically estimates total SC length. The algorithm uses image processing techniques to transform each image into a single-pixel-wide wireframe representation. SC length is then determined by counting the number of pixels in this representation and scaling this value to the level of magnification (see Methods for details). Fig 2 shows two representative images of spermatocytes, one from each cross, and their progression through the image analysis algorithm.

**Fig 3:**
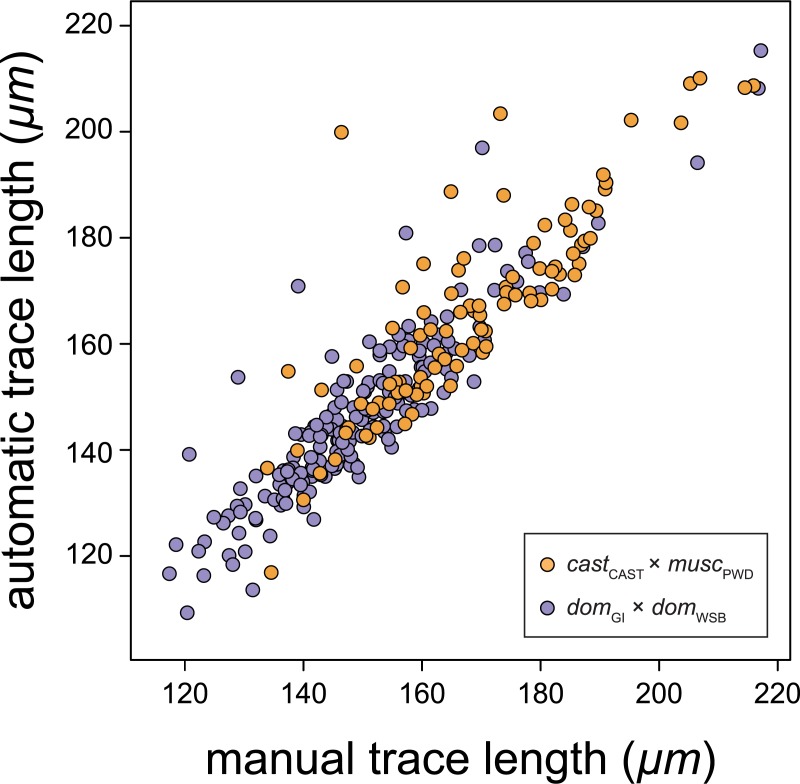
SC lengths from automated versus manual analysis. Each point represents the SC length from a single spermatocyte image in the test set. SC lengths of spermatocytes from *dom*_GI_ × *dom*_WSB_ F_2_ individuals are shown in purple (coefficient of determination, R^2^ = 0.74); those from *cast*_CAST_ × *musc*_PWD_ F_2_ individuals are shown in orange (R^2^ = 0.81).

To assess the accuracy of our algorithm, we first applied it to a set of test images. We randomly selected five F_2_ individuals from each of the two intercrosses and manually measured SC length in the 284 images of spermatocytes from these individuals. These measurements were compared to those produced by the algorithm for the same set of images. SC lengths estimated using our algorithm reliably matched those obtained by manual tracing in our test set for spermatocytes from both crosses (overall R^2^ = 0.83; *cast*_CAST_ × *musc*_PWD_, R^2^ = 0.74; *dom*_GI_ × *dom*_WSB_, R^2^ = 0.81; Fig 3). We also compared the mean SC length of spermatocytes from the same individual and found even better agreement between the algorithm and manual measurements (overall R^2^ = 0.97). Motivated by this high concordance, we applied the algorithm to images of spermatocytes from: 5 *cast*_CAST_, 5 *musc*_PWD_, 289 *cast*_CAST_ × *musc*_PWD_ F_2_s, 4 *dom*_GI_, 6 *dom*_WSB_, and 229 *dom*_GI_ × *dom*_WSB_ F_2_s.

### SC length variation between and within subspecies

*musc*_PWD_ spermatocytes have longer SCs than *cast*_CAST_ spermatocytes (*musc*_PWD_ mean = 174.2 μm, SE = 0.7 μm; *cast*_CAST_ mean = 150.4 μm, SE = 0.4 μm; t-test *p* < 0.05). SC length is continuously distributed among F_2_s from this intercross, with a mean close to the mid-parent value. These characteristics mirror those for the mean number of MLH1 foci in the same set of individuals (S1 Fig and [25]).

*dom*_GI_ spermatocytes have longer SCs than *dom*_WSB_ spermatocytes (*dom*_GI_ mean = 139.6 μm, SE: 1.6 μm; *dom*_WSB_ mean = 131.8 μm, SE: 1.4 μm; t-test *p* < 0.05). Approximately half of the F_2_s from this intercross have mean SC lengths beyond the parental means, a pattern that resembles the distribution of MLH1 foci in the same set of individuals (S1 Fig; [63]).

### Genetic determinants of SC evolution between subspecies

The continuous distributions of mean SC length among spermatocytes from individuals in both intercrosses suggest that SC length is a complex trait controlled by multiple loci. To identify quantitative trait loci (QTL) driving evolution of the SC between and within subspecies, we conducted genome-wide QTL scans using mean SC length as the phenotype.

**Fig 4:**
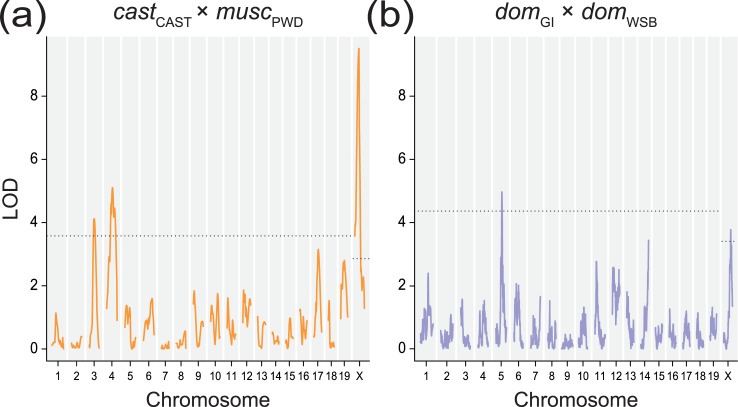
LOD plots for SC length QTL. Plots show significant QTL for SC length in the (a) *cast*_CAST_ × *musc*_PWD_ cross on chromosomes 3, 4, and X and in the (b) *dom*_GI_ × *dom*_WSB_ cross on chromosomes 5 and X. Dashed line shows genome-wide significance level, α = 0.05.

We found three QTL responsible for the SC length difference between *musc*_PWD_ and *cast*_CAST_ on chromosomes X, 3 and 4 (Fig 4A; Table 1). *musc*_PWD_ alleles at QTL on chromosomes 3 and 4 increase mean SC length in an additive manner. In contrast, the *musc*_PWD_ allele at the X-linked QTL decreases SC length, acting in opposition to the phenotypic difference between strains. The summed additive effects of QTL on chromosomes 3 and 4 explain 29.2% of the SC length difference between the two strains. Collectively, the three QTL explain 28.5% of the F_2_ variance. This percentage is substantially less than the 74% of phenotypic variance explained by QTL for MLH1 count in this same cross [25], though variance explained by the SC length QTL may be underestimated as this trait is more difficult to measure (see Discussion).

**Table 1:** QTL for synaptonemal complex length.

*cast*_CAST_ × *musc*_PWD_ cross								
Chr.	Pos. (cM)	LOD	Pos. (Mb)	1.5 LOD Interval (Mb)	% var.^a^	Estimated phenotypic means (SE)^b^		
						*c*_C_*c*_C_ / *c*_C_Y	*c*_C_*m*_P_	*m*_P_*m*_P_ / *m*_P_Y
3	38.0	4.12	103.5	68.0–128.6	6.4	157.8 (1.4)	162.7 (0.9)	165.7 (1.3)
4	44.0	5.10	112.3	65.8–146.5	6.7	158.5 (1.3)	162.0 (0.9)	167.6 (1.7)
X	31.9	9.51	71.7	52.9–90.7	14.6	166.1 (0.8)	—	158.3 (0.8)
*dom*_GI_ × *dom*_WSB_ cross						*d*_G_*d*_G_ / *d*_G_Y	*d*_G_*d*_W_	*d*_W_*d*_W_ / *d*_W_Y
5	44.8	4.97	106.3	102.9–115.4	6.8	139.2 (0.9)	136.5 (0.6)	140.8 (0.8)
X	54.0	3.78	138.1	123.0–153.8	4.9	140.1 (0.6)	—	136.8 (0.6)

^a^ Percentage of phenotypic variance among F_2_s explained

^b^ *c*_C_−*cast*_CAST_; *m*_p_−*musc*_PWD_, *m*_p_Y and *c*_C_Y genotypes for QTL on X

*d*_G_−*dom*_GI_; *d*_W_−*dom*_WSB_, *d*_G_Y and *d*_W_Y genotypes for QTL on X

**Fig 5:**
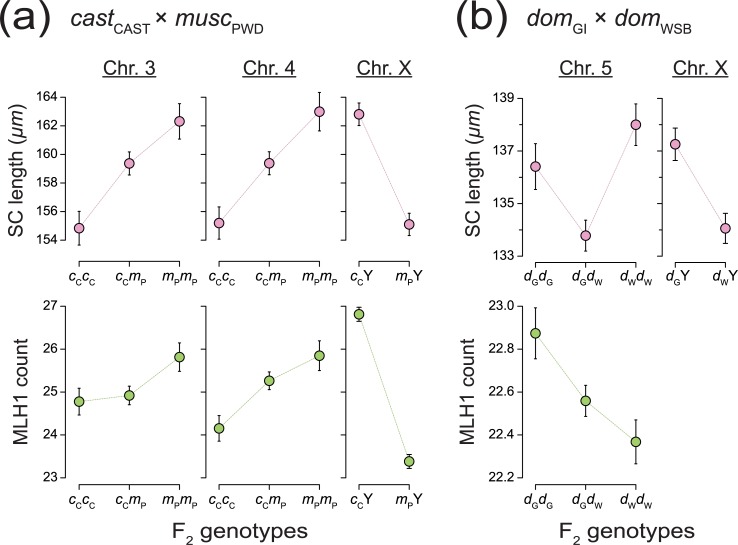
Estimated QTL effects by genotype. Effects on F_2_ individuals estimated at the position of peak LOD on chromosomes with significant QTL for mean SC length (top) and MLH1 foci count (bottom). Error bars show ±1 SE.(a) *cast*_CAST_ × *musc*_PWD_ cross; *c*_C_ – *cast*_CAST_, *m*_P_−*musc*_PWD_ SC QTL: (Chr. 3, 38.0 cM); (Chr. 4, 44.0 cM); (Chr. X, 31.9 cM) MLH1 QTL: (Chr. 3, 66.0 cM); (Chr. 4, 25.0 cM); (Chr. X, 31.9 cM) (b) *dom*_GI_ × *dom*_WSB_ cross; *d*_G_−*dom*_GI_, *d*_W_−*dom*_WSB_ SC QTL: (Chr. 5, 44.8 cM); (Chr. X, 54.0 cM) MLH1 QTL: (Chr. 5, 36.9 cM).

Chromosomes X, 3 and 4 had been found to harbor QTL for mean MLH1 count [25]. The 1.5-LOD intervals of QTL for SC length and MLH1 count overlap on chromosomes X and 4 (Table 2), indicating that a single locus in each of these intervals could affect both traits. Alleles at both of these QTL affect SC length and MLH1 count in similar ways (Fig 5A). In contrast, allelic effects at the chromosome 3 QTL differ for SC length (alleles act additively) and MLH1 count (*cast*_CAST_ allele acts dominantly) (Fig 5A). This difference in phenotypic effects, along with distinct 1.5 LOD intervals, indicates separate QTL on chromosome 3: one locus for SC length (peak LOD = 104 Mb, [68–129 Mb]) and a second, more distal locus for MLH1 count (peak LOD = 150 Mb, 1.5-LOD interval [133–160 Mb]).

**Table 2:** Overlapping QTL.

*cast*_CAST_ × *musc*_PWD_						
Phenotype	Chr.	Pos. (cM)	LOD	Pos. (Mb)	1.5 LOD Interval (Mb)	% var. explained
SC/CO^a^	4	—	—	—	—	—
MLH1 foci	4	25.0	5.07	65.0	48.5–77.0	2.8
SC length	4	44.0	5.10	112.3	65.8–146.5	6.7
SC/CO	7	8.0	6.90	30.3	13.6–47.7	13.1
MLH1 foci	7	11.0	4.77	35.8	6.5–133.9	6.7
SC length^a^	7	—	—	—	—	—
SC/CO	X	34.0	18.65	85.4	71.5–97.5	30.0
MLH1 foci	X	31.9	34.92	71.7	62.3–90.8	45.9
SC length	X	31.9	9.51	71.7	52.9–90.8	14.6
*dom*_GI_ × *dom*_WSB_						
SC/CO	5	47.8	9.19	113.2	104.2–114.6	12.5
MLH1 foci	5	53.5	4.39	124.9	74.3–132.5	6.1
SC length	5	44.8	4.97	106.3	102.9–115.4	6.8

^a^ No significant QTL for phenotype on this chromosome.

### Genetic determinants of SC evolution within subspecies

We discovered two QTL responsible for the SC length difference between *dom*_GI_ and *dom*_WSB_ on chromosomes X and 5 (Fig 4B; Table 1). Together, these loci account for 15.2% of the phenotypic difference between strains (and explain 12.8% of the F_2_ variance). The 1.5-LOD interval for the QTL on chromosome 5 overlaps with a QTL for MLH1 count [63]. QTL effects differ between traits: heterozygotes show reduced SC length compared to homozygotes, whereas alleles act additively to shape MLH1 count (Fig 5B). The X-linked QTL for SC length appears not to affect MLH1 count [63].

None of the QTL for SC length differences within subspecies overlaps with QTL for SC length differences between subspecies.

### Candidate genes for evolution of SC length

To identify candidate genes and mutations for SC evolution, we combined available information from spermatocyte expression data and gene ontology (GO) with the QTL intervals for SC length. We focused on QTL that explained the difference in SC length between *dom*_GI_ and *dom*_WSB_ because of the relatively lower sequence divergence between these strains. We found 301 candidate genes within the 1.5 LOD interval of the QTL on chromosomes X and 5; 11 of these were compelling candidates (table in S1 Data). We filtered the initial candidate list by looking for an association with recombination GO terms (search strings: “recomb”, “synapton”, and “meio”) and increased expression during meiosis in transcriptomic experiments [64,65]. To further refine this list, we considered only those genes with a single nucleotide polymorphism (SNP) between *dom*_GI_ and *dom*_WSB_ that yielded either a nonsynonymous change or a change to a known or inferred transcription factor binding site. We identified two strong candidate genes on chromosome 5 from the whole-genome sequence comparison, *Hfm1* and *Rnf212*, and several candidate mutations within them (Table 3). Variants in both candidate genes have been previously associated with variation in genome-wide recombination rate in mammals [19,26,30,66–68], and both genes are known to be involved in chromosome synapsis [69,70] (see Discussion).

**Table 3:** Candidate genes and mutations for the evolution of SC length within *M*. *m*. *domesticus*.

Gene Symbol	Chr.	Position^a^	Base^b^ Change	AA Change	Genotype in *dom*_GI_ Parents	Variant Effect^c^
Hfm1	5	106853422	T -> C	—	C/C; C/C	Low Info TF site
Hfm1	5	106871789	T -> G	Y -> S	G/G; G/G	SIFT: 0.04
Hfm1	5	106904814	T -> C	T -> A	C/C; C/C	SIFT: 0.74
Hfm1	5	106911653	A -> G	C -> R	G/G; G/G	SIFT: 0.08
Rnf212	5	108729476	T -> C	I -> V	C/C; C/C	SIFT: 0.49
Rnf212	5	108744012	C -> T	—	T/T; T/C	Low Info TF site
Rnf212	5	108770595	C -> T	V -> I	T/T; T/C	SIFT: 0.05

^a^ Position in GRCm38/mm10.

^b^ Base change and amino acid (AA) change in *dom*_GI_ with *dom*_WSB_ as reference.

^c^ Predicted variant effect for nonsynonymous changes as SIFT score 0.0 (deleterious)– 1.0 (tolerated), (Kumar et al. 2009); for variants at predicted transcription factor (TF) binding sites, variants are classified as being in high or low information regions.

### Genetic relationships between SC length and crossover number

**Fig 6:**
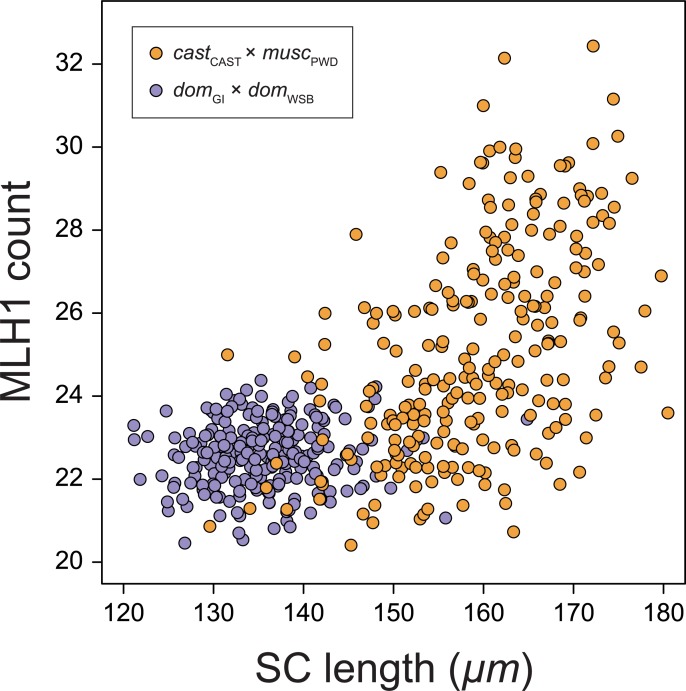
Mean MLH1 foci count and SC length from each cross. Mean MLH1 count and SC length from the same F_2_ individuals. Those from the *cast*_CAST_ × *musc*_PWD_ cross in orange (regression slope (SE): 0.13 (0.01) foci/μm) and from the *dom*_GI_ × *dom*_WSB_ cross in purple (regression slope: 0.01 (0.01) foci/μm).

To examine genetic connections between SC length and crossover number, we compared SC lengths to MLH1 counts obtained for the same spermatocytes [25,63]. SC length and the number of MLH1 foci are positively correlated across spermatocytes in both F_2_ intercrosses (Fig 6). This correlation is significantly stronger in spermatocytes from the *cast*_CAST_ × *musc*_PWD_ cross (Pearson’s r = 0.38, 95% CI = [0.36, 0.40]) compared to those from the *dom*_GI_ × *dom*_WSB_ cross (r = 0.15, CI = [0.12, 0.19]). We examined the ratio of these two phenotypic values from F_2_ individuals in the following analyses to consider the genetic connection between SC length and the number of crossovers. Loci that control this ratio may play a role in controlling crossover interference, the observation from a variety of species that crossovers are spaced more regularly than expected if they occur independently [71].

The ratio of mean SC length to mean MLH1 count (SC/CO ratio) (S3 Fig) varies more among F_2_s from the *cast*_CAST_ × *musc*_PWD_ cross (*cast*_CAST_ mean: 6.5 ± 0.03 μm / focus, *musc*_PWD_: 5.8 ± 0.04, F_2_s: 6.4 ± 0.52) than among F_2_s from the *dom*_GI_ × *dom*_WSB_ cross (*dom*_GI_: 6.0 ± 0.1 μm / focus, *dom*_WSB_: 6.1 ± 0.12, F_2_s: 6.0 ± 0.30 μm / focus). This mirrors the greater divergence of both traits in the inter-subspecific cross. Treating the transformation of the two trait values as an individual trait, we scanned for associated QTL in both crosses.

**Fig 7:**
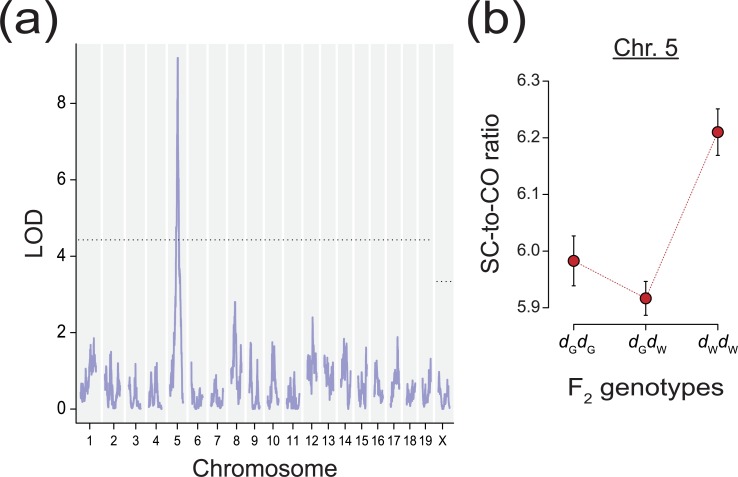
A QTL for the SC/CO ratio in the *dom*_GI_ × *dom*_WSB_ cross. (a) LOD plot for SC/CO ratio shows a significant QTL on chromosome 5. Dashed line shows genome-wide significance level, α = 0.05. (b) Estimated effects of the QTL on chromosome 5 for SC/CO ratio among *dom*_GI_ × *dom*_WSB_ F_2_s by genotype; *d*_G_ –*dom*_GI_, *d*_W_−*dom*_WSB_. Error bars show ±1 SE.

We identified three QTL that influence the SC/CO ratio. These loci overlap with QTL previously identified in single-trait analyses (Table 2; S4 Fig). The interval on chromosome 5 from the *dom*_GI_ × *dom*_WSB_ cross is notable, however. The LOD score for SC/CO ratio at this locus is substantially higher than the score for either of the two traits analyzed separately (Fig 7). Further, this QTL explains 12.5% of the variation in SC/CO ratio among *dom*_GI_ × *dom*_WSB_ F_2_s, substantially more than the 6.1% and 6.8% of variation this interval explains for MLH1 count and SC length, respectively. We interpret these results as evidence that the QTL on chromosome 5 is not only pleiotropic, but responsible for the level of covariation between the two traits. The *dom*_GI_ allele at this locus dominantly reduces the SC/CO ratio among the *dom*_GI_ × *dom*_WSB_ F_2_s (Fig 7B).

### Causal inference of pleiotropic QTL action

To further characterize pleiotropic QTL and identify steps of the recombination pathway they modulate, we performed mediation analysis [72–75]. This approach assesses whether the relationship between two variables exists because of the indirect effect of a third, mediating variable. For example, a genetic interval associated with the number of MLH1 foci may actually be mediated by this interval’s effect on SC length; accounting for the effect of this mediator would reduce or wholly abolish the interval’s association with the number of MLH1 foci.

We tested two models of mediation for each putatively pleiotropic locus—one for each trait acting as a mediator of the other. Mediation was evaluated for each QTL by comparing four regression models of QTL effects on the two traits.

Model 1

QTL → SC length

*Y*_SC_ = β_0_ + β_SC_X_QTL_ + ε

Model 2

QTL → CO count

*Y*_CO_ = β_0_ + β_CO_X_QTL_ + ε

Model 3

QTL → CO count → SC length

*Y*_SC_ = β_0_ + β′_SC_X_QTL_ + α_CO_X_CO_ + ε

Model 4

QTL → SC length → CO count

*Y*_CO_ = β_0_ + β′_CO_X_QTL_ + α_SC_X_SC_ + ε

In this regression framework, β_0_ represents the intercept for each model, ε represents the error term, β_SC_ and β_CO_ are the regression coefficients for the QTL in an unmediated model, β′_SC_ and β′_CO_ are the coefficients for the QTL in a mediated model, and α_SC_ and α_CO_ are the coefficients for each trait when acting as a mediator for the other trait.

We found significant evidence for mediation effects (*p* < 0.01) at QTL on chromosomes 4 and X in the *cast*_CAST_ × *musc*_PWD_ cross (Table 4). Both models of mediation are supported at these loci. The effects of QTL on SC length are significantly mediated by their effects on the number of MLH1 foci, and likewise, the effects of QTL on MLH1 count are significantly mediated by their effects on SC length. We estimated the proportion of QTL effects explained by the mediating variable for each of the two models as

$$f_{SC}=(\beta_{SC}-{\beta \prime }_{SC})/\beta_{SC}$$

$$f_{XO}=(\beta_{CO}-{\beta \prime }_{CO})/\beta_{CO}$$

**Table 4:** Regression coefficients and mediation at pleiotropic loci.

*cast*_CAST_ × *musc*_PWD_											Analytic^a^		Empirical^b^	
Locus (Chr : Pos)	β_SC_	β_CO_	α_SC_	α_CO_	β′_SC_	β′_CO_	*t*_SC_	*t*_CO_	*f*_SC_^c^	*f*_CO_^c^	*p*_SC_	*p*_CO_	*p*_SC_	*p*_CO_
4 : 44.0cM	3.73	0.48	7.2×10^−3^	1.46	2.96	0.24	2.32	3.33	0.21	0.50	0.021	8.7×10^−4^	<1×10^−3^	<1×10^−3^
4 : 25.0cM^d^	3.06	0.48	4.2×10^−3^	1.81	1.96	0.37	2.73	2.63	0.36	0.23	0.006	0.009	<1×10^−3^	<1×10^−3^
X : 31.9cM	-3.67	-1.74	7.2×10^−3^	1.46	-1.08	-1.48	-4.92	-4.18	0.70	0.15	8.7×10^−7^	2.9×10^−5^	<1×10^−3^	<1×10^−3^
X : 33.0cM^d^	-3.38	-1.65	4.2×10^−3^	1.81	-0.36	-1.50	-4.17	-3.22	0.89	0.10	3.1×10^−5^	1.3×10^−3^	<1×10^−3^	<1×10^−3^
*dom*_GI_ × *dom*_WSB_														
5 : 44.8cM	0.80	-0.31	1.8×10^−3^	1.21	1.17	-0.32	-1.99	1.15	-0.46	-0.03	0.047	0.249	0.04	0.08
5 : 36.9cM^d^	0.08	-0.34	9.5×10^−4^	0.80	0.35	-0.34	-1.25	0.13	-3.38	0.06	0.210	0.896	0.19	0.92

^a^ *p* values calculated with two-tailed test using standard normal distribution as the null.

^b^ *p* values calculated by permuting mediating variable among individuals (see Methods).

^c^ Proportion of effects mediated only meaningful for loci that show significant evidence of mediation.

^d^ Coefficients calculated at peak LOD of QTL for MLH1 foci. Coefficients for loci without footnotes calculated at peak LOD of QTL for SC length.

Estimates use a multiple-QTL model for the respective trait (see Methods).

For the QTL on the X chromosome, mediation is much stronger in the model where the QTL effect on SC length is mediated by its effect on the number of MLH1 foci (*f*_SC_ = 0.70 ± 0.28, *f*_CO_ = 0.15 ± 0.09). In contrast, the proportion of effects mediated at the QTL on chromosome 4 is more similar between the two models (*f*_SC_ = 0.21 ± 0.31, *f*_CO_ = 0.50 ± 0.44). We performed analyses on the sensitivity of these findings to differences in measurement error—which can reduce the strength of evidence for mediation—by adding Gaussian noise to the observations. Evidence for mediation and the estimated proportions of effects mediated were found to be negligibly affected by differences in measurement error between the two traits (see Materials and Methods and S1 Methods for details).

## Discussion

We report here the first loci known to be involved in the evolution of SC length, establishing a genetic basis for natural variation in this fundamental meiotic structure. Distinct genomic regions are responsible for evolutionary differences in SC length between and within subspecies of house mice. Several of these loci also control the genome-wide number of crossovers; this pleiotropy partially explains the correlation between SC length and recombination rate widely observed in cytological studies. More broadly, our results demonstrate the power of genetically dissecting natural variation in multiple aspects of the meiotic program to understand differences in recombination rate that exist among organisms.

Our findings point to genetic mechanisms involved in the evolution of SC structure and recombination rate. We took advantage of knowledge of recombination pathways and low levels of sequence variation within *M*. *m*. *domesticus* to nominate strong candidate genes for evolution of the SC. Two genes seem especially worthy of consideration in the chromosome 5 interval that affects SC length, crossover number, and the SC/CO ratio. *Hfm1* (also known as *Mer3*) is a DNA helicase required for the completion of chromosome synapsis and crossover formation in mouse; *Hfm1* knockout mice produce an SC that fails to assemble along the full length of the chromosome axis [69]. Nonsynonymous variants in *Hfm1* have been associated with inter-individual differences in genome-wide recombination rate in cattle and humans [66,68].

*Rnf212* is another strong candidate gene for the chromosome 5 QTL. *Rnf212* is a SUMO ubiquitin ligase that selectively localizes to a subset of recombination sites along the central region of the SC, coupling synapsis to formation of crossover-specific protein complexes [70]. *Rnf212* stabilizes crossover precursors and helps determine whether each recombination site becomes a crossover [70,76]. Variants at *Rnf212* also contribute to inter-individual differences in genome-wide recombination rate in red deer [77], Soay sheep [30], cattle [26,66], and humans [19,22,78,79]. Two amino acid substitutions in *Hfm1* and one in *Rnf212* are predicted to have strongly deleterious fitness effects, suggesting they should be prioritized for further evaluation. The strength of support for these candidate genes, along with the highly specific nature of the phenotypes (SC length and crossover number), should motivate functional testing using genome editing or other approaches.

Joint consideration of SC length and crossover number provided additional clues about the genetic connections between these phenotypes. Models of the relationship between these two traits have concentrated on the order of molecular events surrounding synapsis and recombination [80–82]. We took two approaches to investigating the covariation of SC length and crossover number, focusing on their disparity within and between subspecies. Our first approach treated the ratio of SC length to the number of crossovers as its own trait. Inter-individual differences in this ratio have previously been documented in human spermatocytes [83]. Could upstream control of this ratio be modulating differences in both traits simultaneously? We discovered a locus with such an effect in the *dom*_GI_ × *dom*_WSB_ cross. As expected, loci with a large effect on the SC/CO ratio also affect each trait individually. However, one of the QTL for this ratio (on chromosome 5) had a substantially increased signal—higher LOD, narrower confidence interval, and greater percentage of variance explained—relative to its effect on any single phenotype. These results suggest that the SC/CO ratio is closer to the mechanism of this QTL’s action for both phenotypes. We conclude that SC length and crossover number are causally linked, in part by the action of this locus.

Changes in the length of the SC reflect differences in chromatin packing during meiosis [48–50]. Our observation that the number of crossovers per unit of SC length differs between and within subspecies therefore indicates that the relative spacing of crossovers has evolved in house mice, a species in which crossover interference is strong [84,85]. To our knowledge, the QTL on chromosome 5 is only the second locus suggested to contribute to standing variation in interference (see [31]). The SC has been shown to play a critical role in establishing proper interference [86,87]. A recent model of the SC as a liquid crystal suggests the SC is a conduit for partitioning crossovers by transmitting an interference signal across the chromosome [88].

Our second approach to investigating covariation of SC length and crossover number was to perform mediation analysis on loci that appeared pleiotropic. This approach yielded strong evidence for pleiotropy at QTL on chromosomes 4 and X in the *musc*_PWD_ × *cast*_CAST_ cross. The direction of effects at these loci (longer SC and more crossovers), and their concordance with the overall correlation between the traits, suggests their participation in a singular underlying mechanism. However, the relative magnitude of mediation effects at these two loci indicates that they likely contribute to different elements in the pathway leading to crossover formation. The coordinated variation of SC length, crossover number, and DSBs (along with other molecular intermediaries of recombination) in comparative studies across mammals [57] and mouse strains [89] has been used to suggest that variation in recombination rate is established at the earliest stages of meiosis. Because MLH1 is recruited to crossovers after the SC is completely assembled, the stronger mediation of SC length by crossover number at the X-linked QTL is consistent with an effect on an early decision, perhaps by participating in the crossover/non-crossover decision before synapsis [90,91]. In contrast, the direction of mediation at the QTL on chromosome 4 is consistent with a later action. Stronger mediation of crossover count by SC length at this locus suggests an effect on crossover formation subsequent to the completion of SC assembly, a potential subset of crossovers marked by MLH1 foci [18,92]. Our results indicate that, despite the covariation of these two meiotic phenotypes, natural variation in recombination rates appears to be established at multiple stages in the recombination pathway, at least in mice.

We found that the X chromosome explained variation in SC length both within and between subspecies. No other chromosome had significant QTL from both crosses. Previous studies identified an important role for the X chromosome in the evolution of recombination rate between subspecies of house mice [23,25,28,93]. Our findings extend the contributions of the X chromosome to the evolution of a second meiotic trait connected to recombination: the SC. One theory for the disproportionate role of the X chromosome in recombination rate variation focuses on the possibility of antagonistic sexual conflict [94–96]. If different optima for recombination rates exist between males and females, the X chromosome may accumulate rate modifiers in an evolutionary arms race. Male and female mice also show contrasting patterns of divergence in recombination rate, consistent with such a hypothesis [25,97].

Comparing QTL locations among the two crosses also provides clues about the timescale of SC length evolution and the origins of the causative mutations. The absence of co-localizing QTL between the two crosses is somewhat surprising given the relatively recent divergence of the mouse subspecies [58,98]. We expect some of the genetic variation responsible for differences in SC length should be from alleles that arose before subspecies divergence but that have remained polymorphic within subspecies. A comparison between mapping experiments, as in the crosses presented here, should reveal these ancestrally polymorphic alleles as shared, co-localizing QTL. While our experiments lacked the power to identify more than a handful of SC length QTL, that none were shared may hint at their rapid or recent accumulation. If mutations responsible for SC length divergence are sorted in the lineages we considered, it would imply that they arose during the last few hundred thousand generations (i.e., in the time since the subspecies diverged). Genetic mapping experiments with other strains of house mice would help to pinpoint the temporal origins of the QTL alleles we discovered.

Our conclusions are tempered by a few limitations of our experimental design. The SC is a spatially and temporally dynamic structure [99–101], for which our measurements of length are an incomplete summary. SC length was harder to estimate than crossover count, which complicated joint genetic analysis of the two traits (though our sensitivity analysis suggests robustness of mediation inferences). Since MLH1 foci only mark those crossovers produced by the interference-dependent pathway (the vast majority in mice [18,102]), the connection between SC length and non-interfering crossovers was not accessible with our experimental approach. Because our estimates of SC length were summed across all chromosomes, we could not determine whether each QTL affects multiple chromosomes (in *trans*) or only its own chromosomal region (in *cis*). Nevertheless, we can be certain that the pleiotropic QTL on the X chromosome acts in *trans* since our crossover measurements were restricted to autosomes in male meiotic cells. Meiosis and recombination in females may operate differently. While QTL mapping is a powerful approach to identify loci responsible for variation, its resolution is limited by the number of recombinant offspring and analyses tend to overestimate the effect size of significant loci [103–105]. Higher mapping resolution could reveal that some of our apparently pleiotropic QTL are instead composed of multiple, closely linked loci.

This first genetic portrait of natural variation in SC length raises key questions about the evolution of this fundamental meiotic trait. How is SC length related to fitness? Does natural selection target SC length through its effects on meiotic chromatin organization, recombination rate, or some other trait? Does divergence of SC length constrain or accelerate recombination rate divergence? Our findings should motivate incorporation of SC length into comparative studies of recombination rate evolution as well as genetic dissections of shifts in the underlying pathways.

## Materials and methods

### Ethics statement

All animal care and experimental protocols were approved by the University of Wisconsin Animal Care and Use Committee (Protocol #M005388, #V005209). Laboratory mice were euthanized by trained personnel via CO_2_ inhalation.

### Crosses

Data presented in this study was gathered using images of spermatocytes from F_2_ males from two separate intercrosses. In the *musc*_PWD_ × *cast*_CAST_ cross, 315 F_2_ males were sacrificed at approximately 10 weeks of age; 289 were included in the final analysis. The second cross, *dom*_GI_ × *dom*_WSB_, included 315 F_2_ males sacrificed at approximately 16 weeks of age; 229 were included in the final analysis. Additional details on cross design and animal husbandry can be found in [25] and [106].

### Tissue collection, immunostaining, and microscopy

Details on the preparation of spermatocyte spreads and immunostaining can be found in [63] and [25]. Here, we briefly summarize the shared steps taken to arrive at stained spermatocyte images.

Seminiferous tubules were extracted from the testis of sacrificed males and incubated in hypotonic buffer. The macerated tubules were then ripped apart to liberate spermatocytes. The cellular slurry was fixed onto a glass slide with a paraformaldehyde solution and allowed to dry. These prepared slides were incubated with primary antibodies against MLH1, a mismatch repair protein that localizes to sites of meiotic crossover, and SYCP3, an essential structural element spanning the synaptonemal complex. After several wash steps, the slides were then incubated with a set of secondary antibodies tagged with fluorophores, at 488 nm and 568 nm for MLH1 and SYCP3 respectively, and then mounted for visualization.

Slides from the *musc*_PWD_ × *cast*_CAST_ cross were imaged on a Zeiss Axioskop microscope with an AxioCam HRc camera. Images from this cross were stored as .tiff files with a resolution of 1030×1300 pixels and 150 pixels per inch. Slides from the *dom*_GI_ × *dom*_WSB_ cross were imaged on a Zeiss Axioplan 2 microscope with an AxioCam HR3 camera. Images from this cross were stored as .tiff files at either 1030×1300 or 1388×1040 at 150 pixels per inch. In both cases, images were captured with a 100× objective lens.

### Measuring SC length

All images were manually curated and only cells with a clearly condensed, full set of 20 bivalents were included. Images of cells with obvious defects or damage from handling were omitted.

We utilized techniques from computer vision to determine the total length of the SC in each spermatocyte from captured immunofluorescent images. We applied algorithms for image processing and analysis as implemented in the scikit-image package for Python 3 (scikit-image.org [107]). For each image, we first isolated the red channel, which contains information from the fluorescence of secondary antibodies against anti-SYCP3 at 568 nm. The image gradient on the isolated channel was then taken with a 3 pixel-wide disk structuring element. This creates an image where regions of contrast, or edges, are enhanced. Otsu’s method [108] was applied to the image gradient, a clustering technique on pixels that reduces the grayscale gradient image to a binary image. Spurious pixels were removed by applying a morphological opening operator, followed by a morphological closing, with a 4 pixel-wide square structuring element. Finally, the cleaned binary image was reduced to a single-pixel wide representation with the skeletonize algorithm [109] implemented in scikit-image. The total number of pixels in this single-pixel wide representation was taken as the total SC length for a spermatocyte.

The reliability of this technique was assessed by comparison to measurements made by manual tracing. Performance was evaluated in a test set of 217 spermatocyte images, from 5 *musc*_PWD_ × *cast*_CAST_ F_2_s and 5 *dom*_GI_ × *dom*_WSB_ F_2_s.

### Genotyping

Mice from the *musc*_PWD_ × *cast*_CAST_ cross were genotyped at 295 SNPs using the Sequenom iPLEX MassARRAY system [25,110]. Of these, 222 SNPs with Mendelian segregation patterns were retained for the QTL analysis. These markers were monomorphic for different alleles in the parents and consistent with the expected genotypic ratio of 1:2:1 among F_2_s. Mice from the *dom*_GI_ × *dom*_WSB_ cross were genotyped at 77,808 markers on the Mega Mouse Universal Genotyping Array (MegaMUGA [32,111]). Of these, 11,833 SNPs with Mendelian segregation patterns were retained for the QTL analysis.

### QTL analysis

All QTL analyses were performed in R (v. 3.3.3) [112] with the R/qtl package (v. 1.40–8) [113]. Representative total SC length for each individual was calculated by taking the mean SC length among spermatocyte images from that individual. Individuals were represented by a median of 18 spermatocyte images and those represented by fewer than 5 images were omitted from the analysis. Haley-Knott regression [114] was performed on data from both crosses to identify QTL for variation in mean SC length. Individuals were weighted by the number of spermatocyte observations, and cross direction was included as an additive covariate. Thresholds for significance were determined by permutation, with genome-wide α = 0.05, and established from 1000 replicates for each cross. Phenotypic means and allelic effects for QTL were estimated at the position of peak LOD with the effectplot function in R/qtl. Percent variance explained by each QTL was estimated under a multiple-QTL model, including all significant intervals from single-QTL scans for each respective trait, using the fitqtl function. We tested multiple QTL models by applying a forward/backwards stepwise search algorithm with penalized LOD scores [115], implemented using the stepwise function in R/qtl. We also evaluated models including epistasis between detected QTL in both intercrosses but did not find any evidence for additional QTL or interactions between detected QTL.

### Identification of candidate genes for SC length variation

Candidate genes fell within the 1.5 LOD interval of QTL peaks identified in the *dom*_GI_ × *dom*_WSB_ cross. We included only genes that were associated with a GO term that included the search strings: “recomb”, “synapton”, or “meio” [116]. We further narrowed candidates by focusing on genes with evidence of increased expression in meiosis leading up to mid-pachytene. Temporal expression patterns were assembled from two RNA-sequencing data sets that staged transcript abundance in mouse spermatocytes [64,65]. Candidate mutations within genes were then identified by inspecting SNPs between *dom*_GI_ and *dom*_WSB_. The full set of SNPs between strains was determined by whole-genome sequencing of the *dom*_GI_ parents to an average depth of 10× and comparison to the *dom*_WSB_ genome [117]. Further details on sequencing and variant identification can be found in [63]. Variants 1kb upstream of the transcription start site, as annotated by the UCSC genome browser on the GRCm38/mm10 build, were included when evaluating potential changes to transcription factor binding sites. Finally, we used the Ensembl Variant Effect Predictor [118] to characterize the predicted fitness consequences of the mutations and included in the final list only those candidates that resulted in a nonsynonymous change or alteration to a transcription factor binding site.

### Mediation analysis

Regression coefficients for tests of mediation were calculated from the estimated effect of the locus in question under a multiple-QTL model. All significant QTLs for a given trait were fit in a regression with and without the mediating trait as a covariate, implemented with the fitqtl function in R/qtl. These same QTLs were also fit in a regression of the mediating trait without the other trait as a covariate. The full model for the regression of each trait can be expressed generally as

$$Y=\beta_{0}+\sum_{i}(\beta_{a,i}+\beta_{d,i})X_{{QTL}_{i}}+\alpha X_{cov}+\varepsilon$$

where QTL_*i*_ is the *i*-th QTL in the model, β_a,*i*_ and β_d,*i*_ are the estimated additive and dominance effects for that QTL, and *X*_cov_ is the mediating variable. At the locus of interest, these coefficients were estimated at the position of peak LOD for each of the regression models. Our assessment of mediation at each locus was limited to its additive effect, estimated by the coefficient β_a,*i*_, corresponding to half the difference between the trait means of the two homozygotes. The covariate’s effects, α, are consistent within each multiple-QTL model. Because a different multiple-QTL model exists for each trait, with different peak LOD positions, we tested for evidence of mediation under both; the results were similar (Table 4).

We used the Sobel test to evaluate the significance of the mediation effect in each model [72–75]. The test statistic was calculated in the two mediation models as

$$t_{SC}=(\alpha_{CO}\beta_{CO})/{SE}_{CO}$$

$$t_{CO}=(\alpha_{SC}\beta_{SC})/{SE}_{SC}$$

where the SE is the pooled standard error,

$$SE=\sqrt{\alpha^{2}\sigma_{\beta }^{2}+\beta^{2}\sigma_{\alpha }^{2}}$$

with regression coefficients, α and β, and their variances, σ_α_^2^ and σ_β_^2^, for each model respectively. The standard normal distribution is typically used as the null distribution for the Sobel test statistic, and the analytic *p*-values reported are two-tailed tests against this null. The normality of the test statistic was also assessed by permuting the mediating variable among individuals within each cross and recalculating the regression coefficients. This was accomplished by resampling the value of the mediating trait without replacement, effectively shuffling the covariate values among the F_2_ individuals within each cross. The regressions were then performed on the shuffled values of *X*_cov_, producing permuted coefficients for the test statistic. This procedure was repeated 10,000 times for each locus. These permutations showed that *p*-values calculated from these empirical distributions were consistent with the analytic results, and that the analytic test was conservative in several cases (S5 Fig; note lower kurtosis).

The reported proportions of effects mediated were estimated using coefficients from a multiple-QTL model of SC length. The ± 1 standard errors for these values were calculated by propagating the error from estimates of the regression coefficients. We also analyzed the sensitivity of these estimates to differences in measurement error between the two traits. This was done by adding Gaussian noise to observations from spermatocytes until the expected within-individual coefficient of variation was identical for both traits (S6 Fig; see S1 Methods for details). The proportion of effects mediated was recalculated in 1,000 replicates with artificial noise. The effect of this noise on the estimates was found to be much smaller than the standard error calculated from the regression coefficients.

## Supporting information

S1 Fig Histograms of SC length and MLH1 count from individual spermatocytes by cross. Vertical bars, in red, indicate parental values for each cross.
F_2_ mean (SD) for SC length and MLH1 count, *cast*_CAST_ × *musc*_PWD_: 159.0 (18.7) μm, 25.2 (3.3) foci; *dom*_GI_ × *dom*_WSB_: 135.7 (13.4) μm, 22.6 (1.7) foci.
Parental values for SC length and MLH1 count, *cast*_CAST_: 150.4 μm, 21.8 foci; *musc*_PWD_: 174.2 μm, 29.9 foci; *dom*_GI_: 139.6 μm, 22.6 foci; *dom*_WSB_: 131.8 μm, 22.3 foci.
(EPS)

S2 Fig Histograms of mean SC length and MLH1 count from F_2_ individuals by cross. Vertical bars, in red, indicate parental values for each cross.
F_2_ mean (SD) for SC length and MLH1 count, *cast*_CAST_ × *musc*_PWD_: 159.1 (9.7) μm, 25.1 (2.6) foci; *dom*_GI_ × *dom*_WSB_: 135.5 (6.6) μm, 22.6 (0.8) foci.
Parental values for SC length and MLH1 count, *cast*_CAST_: 150.4 μm, 21.8 foci; *musc*_PWD_: 174.2 μm, 29.9 foci; *dom*_GI_: 139.6 μm, 22.6 foci; *dom*_WSB_: 131.8 μm, 22.3 foci.
(EPS)

S3 Fig Histogram of SC/CO ratio by cross. Vertical bars, in red, indicate parental values for each cross.
F_2_ mean (SD) for SC/CO ratio, *cast*_CAST_ × *musc*_PWD_: 6.4 (0.52) μm / focus, *dom*_GI_ × *dom*_WSB_: 6.1 (0.12) μm / focus.
Parental values for the SC/CO ratio in *cast*_CAST_: 6.5 μm / focus, *musc*_PWD_: 5.8 μm / focus, *dom*_GI_: 6.0 μm / focus, and *dom*_WSB_: 6.1 μm / focus.
(EPS)

S4 Fig LOD plot of SC-to-MLH1 ratio in the *cast*_CAST_ × *musc*_PWD_ cross. Plot shows significant QTL on chromosomes 7 and X. Dashed line shows genome-wide significance level, α = 0.05.
(EPS)

S5 Fig Normal quantile-quantile plots of Sobel test statistic from permutation analysis. Test statistics calculated from regression coefficients estimated at peak LOD of each QTL for SC length among 10,000 permutations. Several distributions appear platykurtic, but this is conservative for the analytic test of significance.
(EPS)

S6 Fig Intraindividual variation of recombination phenotypes. Boxplots of the coefficient of variation for MLH1 count and SC length among F_2_ individuals organized by cross. Individuals from the *cast*_CAST_ × *musc*_PWD_ cross, in orange, have greater variation for both traits than those from the *dom*_GI_ × *dom*_WSB_ cross, in purple.
(EPS)

S1 Methods Measurement error in mediation analysis. (PDF)

S1 Data Archive of raw data, candidate gene tables, and source code. (ZIP)
