# Supplementary material for: A first genetic portrait of synaptonemal complex variation
Source: PLoS Genet. 2019 Aug 26;15(8):e1008337. doi: 10.1371/journal.pgen.1008337 (PMC6730954; doi:10.1371/journal.pgen.1008337)
Supplement: S1 Methods — (PDF) [file pgen.1008337.s007.pdf]

S1 Methods

Measurement error in mediation analysis

The effect of the X chromosome QTL on SC length was much more strongly mediated by its effect on the number of MLH1 foci ($f_{SC} = 0.70 \pm 0.28$, $f_{CO} = 0.15 \pm 0.09$).
where,

$$f_{sc} = (\beta_{SC} - \beta'_{SC})/\beta_{SC}$$

$$f_{xo} = (\beta_{CO} - \beta'_{CO})/\beta_{CO}$$

A potential explanation for this disparity is greater measurement error in SC length. If a great deal of error exists in measurements of SC length, a true mediation effect from QTL → SC length → CO count could be obscured, reducing our estimate of f_{CO}.

We were interested in reanalyzing the mediation effect and comparing estimates for the proportion of effect mediated, f, in light of the potential for greater measurement error in SC length.

To compare the measurement error between the two traits, we first examined the variance within each individual. Two immediate issues are apparent: first, they do not have the same dimensions and second, there is significant heteroscedasticity.

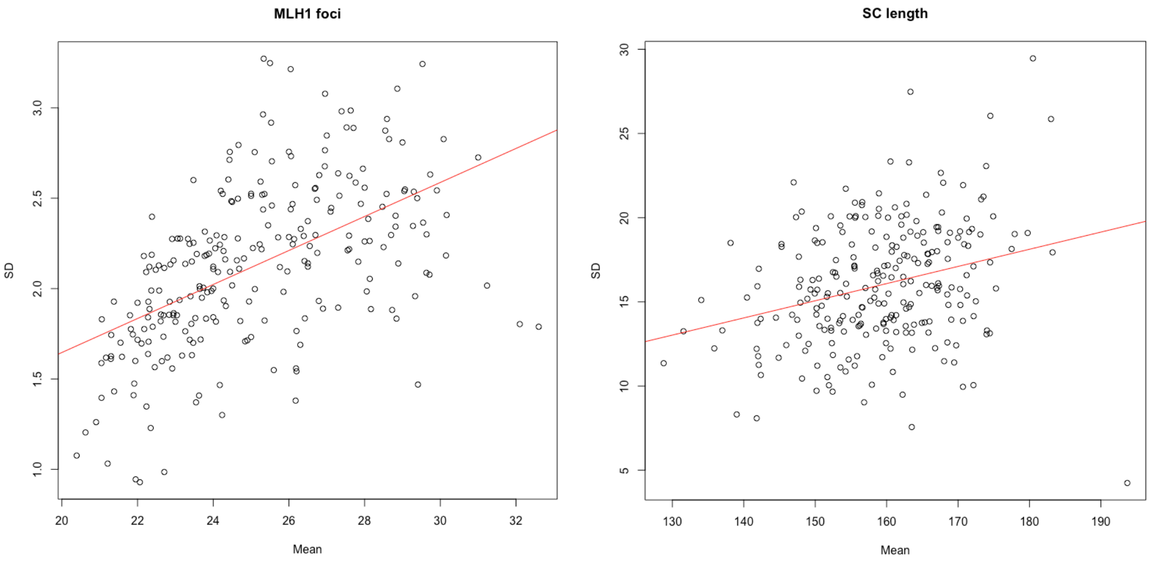

$R^2 = 0.28$ $R^2 = 0.08$

Within-individual heteroscedasticity for MLH1 foci and SC length.
Each point represents the values (mean and standard deviation) from one individual.

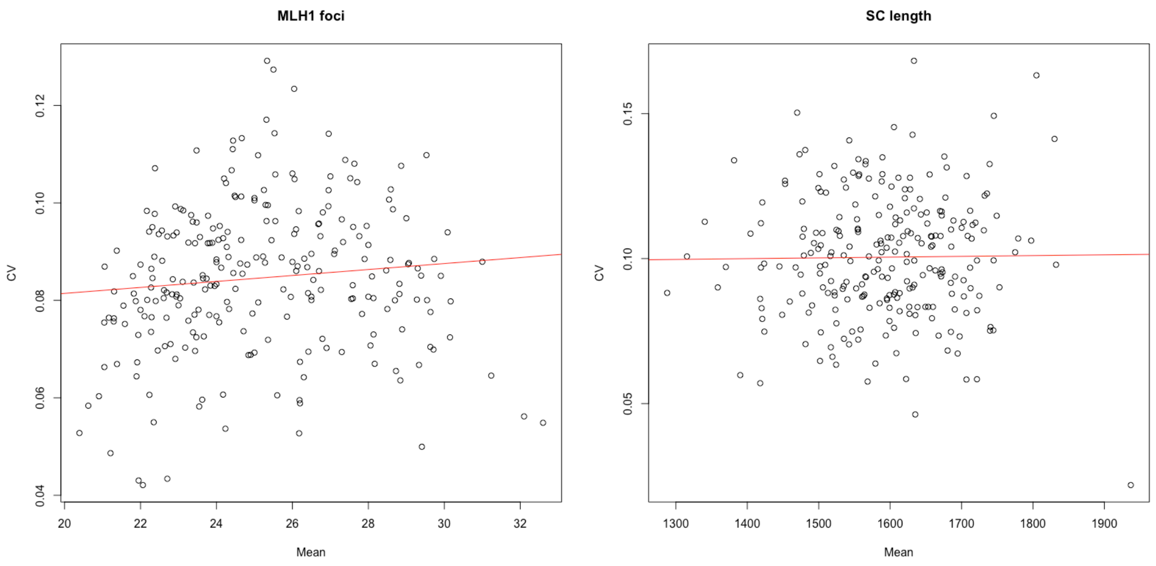

$R^2 < 0.01$ $R^2 < 0.01$

CV describes within-individual variation without heteroscedasticity.

By using the coefficient of variation (CV), we can see that the within-individual variation for SC length is greater than for MLH1 foci. Some of this may be due to greater measurement error for SC length, but we cannot tell how much.

A conservative estimate would be to assume that all of the difference in CV is due to measurement error. Thus, we add random noise to measurements of MLH1 foci from each spermatocyte until its within-individual CV matches that observed for SC length.

Let the mean observed difference between the CV of SC length and MLH1 foci be denoted as δ_{CV}. The noise we add for individual *i* shall be normally distributed as,

$$\varepsilon_i \sim N(0, \sigma_{\varepsilon_i}^2)$$

Let the observed number of MLH1 foci for individual *i* be denoted as Y_{CO}, with mean μ_{CO} and variance σ^2_{CO}.

Because variance is additive as,

$$\mathrm{Var}(Y_{CO} + \varepsilon_i) = \sigma_{CO}^2 + \sigma_{\varepsilon_i}^2$$

the variance for ε_i, such that the CV for MLH1 measurements is increased by δ_{CV}, can be calculated from

$$\frac{\sigma_{CO}}{\mu_{CO}} + \delta_{CV} = \frac{\sqrt{\sigma_{CO}^2 + \sigma_{\varepsilon_i}^2}}{\mu_{CO}}$$

Below, a plot of the within-individual mean and CV for MLH1 foci after the addition of random noise.

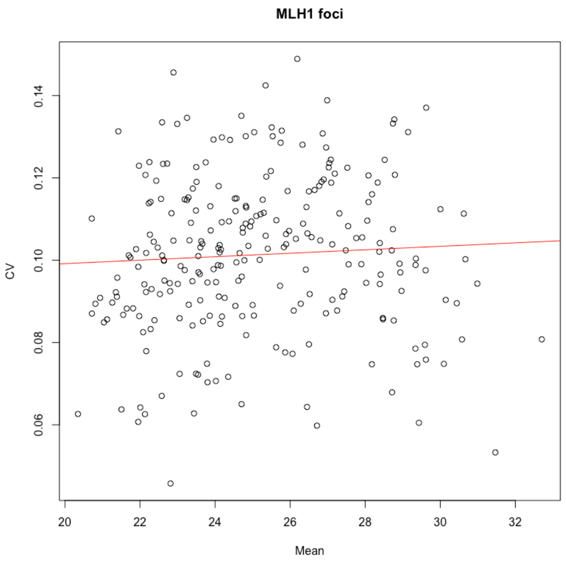

The mediation analysis is repeated on the data with artificial noise, yielding new values for f_{SC} and f_{CO}. Below, the results from 1000 replicates of adding random noise and reanalysis.

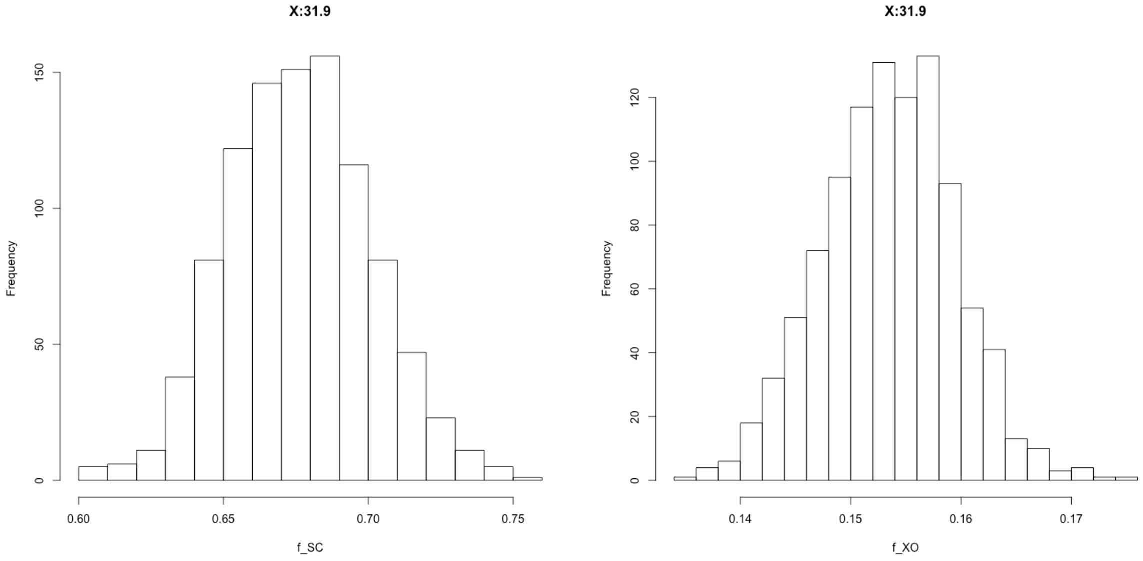

1000 replicates with noise:
mean f_{SC} = 0.676
mean f_{CO} = 0.154

The mean value for f_{SC} is lower than observed in the actual data, as expected from the addition of noise to MLH1 count, but the difference is negligible. The standard error associated with estimating effect size and QTL position completely overwhelms the potential effects of measurement error.
